# Supplementary material for: Comparing the Quality of Primary Care between Public and Private Providers in Urban China: A Standardized Patient Study
Source: Int J Environ Res Public Health. 2021 May 11;18(10):5060. doi: 10.3390/ijerph18105060 (PMC8151428; doi:10.3390/ijerph18105060)
Supplement: Supplementary file 1 [file ijerph-18-05060-s001.zip › ijerph-1195351-supplementary.pdf]

Table S1. Checklist of unstable angina and asthma.

Unstable angina		Asthma
• Pain location<br>• Type of pain<br>• Shortness of breath<br>• Pain radiation		• Onset of problem<br>• Cough<br>• Trigger<br>• Constant or episodic<br>• Duration
• Age<br>• Pain start time (this episode)<br>• Similar pain previously<br>• When started having pain previously<br>• Pain severity<br>• Activity when pain started		• Age<br>• When started having difficulty breathing<br>• Problem in childhood<br>• Frequency
• Smoking<br>• Drinking alcohol<br>• Family history		• Smoking<br>• Family history
• Sweating<br>• Nausea<br>• Acid reflux or stomach<br>• Constipation<br>• Normal stool<br>• Diarrhea		• Expectoration<br>• Fever<br>• Chest pain<br>• Weight loss<br>• Sweating
• Blood pressure<br>• Pulse<br>• Auscultation<br>• Temperature		• Auscultation<br>• Blood pressure<br>• Pulse<br>• Temperature
• Electrocardiogram		-

Table S2. Evaluation criteria for Unstable Angina and Asthma.

	Unstable Angina	Asthma
Adherence to the checklist	1. The proportion of recommended questions asked in the checklists<br>(1) The proportion of essential questions asked in the checklists<br>(2) The proportion of important questions asked in the checklists<br>(3) The proportion of risk factors asked in the checklists<br>(4) The proportion of accompanying symptoms asked in the checklists<br>2. The proportion of recommended examinations asked in the checklists<br>(1) The proportion of physical examinations performed in the checklists<br>(2) The proportion of laboratory examinations performed in the checklists	
Accuracy of diagnosis	1. Correct diagnosis<br>Angina; Myocardial infarction<br>2. Partially correct diagnosis<br>Heart disease<br>3.Incorrect diagnosis (provided by primary care providers)<br>Myocardial ischemia, neuralgia, menopausal syndrome, myocarditis, cervical spondylosis, chest congestion, swelling in chest.	1. Correct diagnosis<br>Asthma<br>2. Partially correct diagnosis<br>Allergies; Breathing problem<br>3.Incorrect diagnosis (provided by primary care providers<br>Heart disease, cold, coronary heart disease, respiratory infection, myocardial ischemia, acute pharyngitis, mycoplasma infection.
Appropriateness of treatment	1. Correct treatment<br>Aspirin, clopidogrel/ or other anti-platelet agents, referral.<br>2. Unnecessary or harmful (provided by providers) Antibiotics, ginkgo oral liquid, Naoxintong capsule, oral electrolyte solution, psychiatric medication, theophylline	1. Correct treatment<br>Bronchodilators, theophylline, inhaled or oral corticosteroids, leukotriene inhibitors, inhaled anticholinergic<br>2. Unnecessary or harmful (provided by providers)<br>Aspirin, compound liquorice tablet, erythromycin, lung-nourishing semifluid extract, ginkgo oral liquid, cephalosporin

Table S3. Scoring methods of PCC.

PCC		Scoring	Source
1. Exploring disease and illness experience			
1.1 *Exploring disease experience*			
1.1.1	Number of recommended questions asked	1 if physician asked the recommended question, 0 otherwise. Calculate the total score of all the recommended items.	SP checklist
1.1.2	Number of recommended exams performed	1 if physician preformed the recommended exam, 0 otherwise. Calculate the total score of all the recommended items.	SP checklist
1.1.3	Number of essential questions asked	2 if physician asked the essential question, 0 otherwise. Calculate the total score of all the essential items.	SP checklist
1.1.4	Number of essential exams performed	2 if physician preformed the essential exam, 0 otherwise. Calculate the total score of all the essential items.	SP checklist
1.2 Exploring illness experience			
1.2.1	The physician made you feel free that you were willing to show your symptoms and fears	Using 5-point Likert scale (e.g. strongly agree, agree, uncertain, disagree, strongly disagree); ranging from 5 to 1.	SP exit survey
2. Understanding the whole person			
2.1	Area of family	1 if physician asked area of family, 0 otherwise.	SP checklist
2.2	Family history	1 if physician asked family history, 0 otherwise.	SP checklist
2.3	Employment	1 if physician asked employment, 0 otherwise.	SP checklist
3. Finding the common ground			
3.1	You agreed with the physician's opinion about the problem	Using 5-point Likert scale (e.g., strongly agree, agree, uncertain, disagree, strongly disagree); ranging from 5 to 1.	SP exit survey
3.2	The physician fully explained this problem to you	Using 5-point Likert scale (e.g., strongly agree, agree, uncertain, disagree, strongly disagree); ranging from 5 to 1.	SP exit survey
3.3	The physician fully explained the treatment plan to you	Using 5-point Likert scale (e.g., strongly agree, agree, uncertain, disagree, strongly disagree); ranging from 5 to 1.	SP exit survey
3.4	Whether the physician gave advice to discuss your roles in disease management	1 if physician gave advice to discuss patient's roles in disease management, 0 otherwise.	SP exit survey
3.5	Overall, do you like this physician	1 if patient likes this physician, 0 otherwise.	SP exit survey
